# *In Vitro* Bioaccessibility Protocol for Chlorophylls

**DOI:** 10.1021/acs.jafc.1c02815

**Published:** 2021-07-30

**Authors:** Isabel Viera, Marta Herrera, María Roca

**Affiliations:** Group of Chemistry and Biochemistry of Pigments. Food Phytochemistry Department, Instituto de la Grasa, Consejo Superior de Investigaciones Científicas (CSIC), University Campus, Building 46, Carretera de Utrera km. 1, Sevilla 41013, Spain

**Keywords:** bioaccessibility, chlorophylls, *in vitro* digestion, micellarization, vegetable puree, virgin olive oil, fruit juice, pheophorbide, pheophytin, chlorin, pyropheophytin

## Abstract

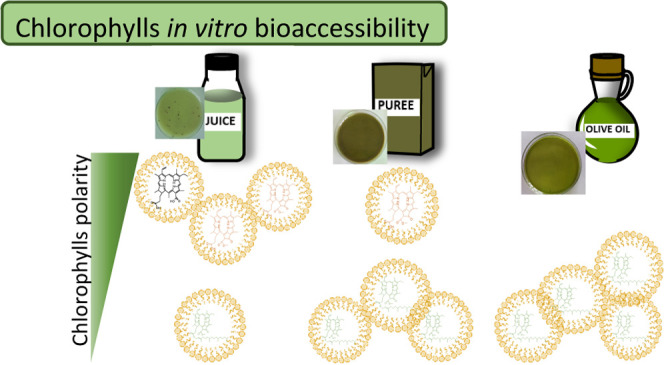

The daily ingestion of chlorophylls has been estimated at 50 g, but the knowledge about their bioaccessibility is limited. Different *in vitro* models have been utilized to estimate their potential bioavailability, but among other factors, the diversity of structures, chemical properties, and lability of chlorophylls hamper the investigations. By the first time, three extreme food matrices, one rich in fiber (vegetable puree), one rich in fat (virgin olive oil), and one liquid (fruit juice), have been assayed for chlorophyll bioaccessibility, controlling crucial variables. Chlorophyll polarity and food matrix were the determining factors, but surprisingly, chlorophyll bioaccessibility was affected during the application of the *in vitro* standardized protocol. Therefore, the present research has identified the reactions that can be biased during the estimation of chlorophyll bioaccessibility, defining a specific protocol in the function of chlorophyll structures.

## Introduction

Chlorophylls are lipophilic pigments responsible for the green pigmentation in photosynthetic organisms: plants, seaweeds, microalgae, *etc*., and, therefore, the most abundant pigments on earth. But besides their primary function in photosynthesis, chlorophylls have shown a large variety of biological activities, including antimutagenic effect, antigenotoxic properties, and a potent antioxidant capacity to scavenge free radicals, preventing lipid oxidation.^1,2^ Chlorophylls are a daily part of our diet, not only with the consumption of fresh fruits and vegetables, seaweeds, *etc*. but also with the ingestion of processed food containing authorized green food colorants as copper and noncopper chlorophylls and “chlorophyllins”: E140 and E141.^3^ However, the available information about chlorophyll bioaccessibility and bioavailability is limited. This may be due to the fact that they were considered to be nonabsorbable by the human body^4^ for a long time. However, in the last few decades, few studies have reported the *in vivo* assimilation of chlorophylls,^5,6^ and more recently, the existence of a clear first-pass metabolism and systemic assimilation has been described,^3,7^ depending on the nature of the chlorophyll compound.

Nevertheless, further studies are needed to understand in-depth the bioavailability of chlorophylls, as we are at the beginning, and *in vitro* models are the current method of investigation since they are faster, less expensive, have no ethical implications, allow the possibility to study a higher number of variables and have shown consistent results in comparison to *in vivo* trials carried out with chlorophylls in humans and animals.^8,9^ Simulated digestion methods typically comprise the oral, gastric, and small intestinal phases. These methods try to mimic physiological conditions, taking into account the presence of digestive enzymes and their concentrations, pH, digestion time, and salt concentrations, among other factors.^8,10^ Although there are several *in vitro* digestion methods available in the bibliography, the mentioned factors show significant variation among themselves, which has not allowed worldwide comparison of results across different research groups.^11^

Several works can be found in the bibliography where *in vitro* digestion of chlorophylls is carried out. Most of the pioneer studies were based on Miller^12^ and Garret^13^ methods,^4,15−17^ but the most recent research studies apply the INFOGEST protocol.^18−20^ Such methodology has been recently updated to INFOGEST 2.0.^21^ The protocol represents a proposal for assay standardization based on physiological parameters, improving the comparability between different studies and consequently the development of practical conclusions about nutrition in human health.^22,23^ However, the protocol is not specific for any compound and needs to be specifically adapted to each food component.^23^ To date, the INFOGEST method has been applied to evaluate the bioaccessibility of different compounds, probiotics,^24^ prebiotics,^25^ cholesterol, carotenoids,^26^ carbohydrates,^27,28^ and proteins,^29^ among others.

Chlorophyll is the generic name that compiles more than 100 different chlorophyll structures, gathering different structural rearrangements, chemical behavior, polarity, *etc*.^30^ The complexity increases as the chlorophylls present in foods suffer from chemical/biochemical modifications due to natural ripening/senescence, processing, and storage. In addition, new chlorophyll structures can be present in foods included as part of the authorized green food colorants. Taking into account such an array of compounds and the inherent lability of chlorophylls, it is essential to analyze in detail the impact of the different phases of the bioaccessibility assay to know the goodness of the methodology when working with chlorophylls and its limitations exactly. We have assumed a large variety of different variables applying different *in vitro* bioaccessibility models without a consensus regarding chlorophyll studies, such as extraction method, type of mixing, centrifugation speed, the influence of filtering, or utilization of gastric lipase. The aim of this study is to establish a specific protocol to determine *in vitro* chlorophyll bioaccessibility accurately, defining the methodology depending on the chlorophyll composition of the food matrix.

## Material and Methods

All of the following procedures were carried out under green light to avoid the photo-oxidation of chlorophyll pigments.

### Chemicals and Reagents

Human α-amylase, porcine pepsin, porcine bile, porcine pancreatin, salts, and the chemicals needed for enzymatic determinations were supplied by Sigma-Aldrich Chemical Co. (Madrid, Spain). Gastric lipase (RGE15) was provided by Lipolytech (Marseille, France). high performance liquid chromatography (HPLC)-grade solvents (acetone, methanol) were supplied by VWR BDH Chemicals (Radnor), except for *N*,*N*-dimethylformamide (DMF), which was supplied by Scharlab (Barcelona, Spain). The purified water was obtained from a Milli-Q water purification system (Millipore, Milford, MA). Chlorophyll *a* and *b* and pheophytin *a* were purchased from Sigma-Aldrich Chemical Co., and chlorin e_4_ and rhodin g_7_ were acquired from Frontier Sci (Utah). The rest of chlorophyll standards were laboratory-produced following established protocols.^31,32^

### Samples

The study was carried out with three different food matrices, a liquid aqueous detox juice, a green vegetable puree, and virgin olive oil. The apple juice contained less than 0.5 g of fat, 4.8 g of sugars, and 0.2 g of proteins per 100 mL, while virgin olive oil was 100% fat. The pea/broccoli puree (1:1) had 2.8 g of fiber, 0.3 g of fat, and 3.6 g of carbohydrates per 100 g. All of them were bought at a supermarket and stored following manufacturer instructions until their analysis was in triplicate.

### Standard *In Vitro* Bioaccessibility Assay

Unless the contrary was specified, the conditions for the *in vitro* digestion and micellarization were developed following the static INFOGEST *in vitro* digestion protocol,^21^ which comprised oral, gastric, and intestinal phases. Frozen electrolyte solutions of digestion fluids were tempered, and CaCl_2_(H_2_O) and the necessary enzyme dilutions were added immediately before use. A preliminary experiment with each food was run to adjust the volume of HCl and NaOH necessary to reach the required pH. Enzyme activities were determined following the protocols described in Brodkorb et al.^21^ and the help of the cited videos. Three replicates of the *in vitro* digestion procedure were carried out for each sample. The initial food samples for the oral phase consisted of 5 g of puree or juice and 5 g of an oily mixture (0.4 g virgin olive oil + 4.6 g water). These samples were mixed with 4 mL of warmed simulated salivary fluid (SSF) electrolyte stock solution (pH adjusted to 7.0 with NaOH), 25 μL of 0.3 M CaCl_2_ (H_2_O)_2_, 25 μL of α-amylase (1000 U/mL, 0.625 g/10 mL SSF), and ultrapure water to a final volume of 10 mL. The samples were incubated for 2 min at 37 °C in a rocker shaker (VWR Rocking platform, at 85 rpm). For the gastric phase, the oral bolus was mixed with 8 mL of previously warmed simulated gastric fluid (SGF) electrolyte stock solution and 5 μL of CaCl_2_ (H_2_O)_2_ 0.3 M, adjusting the pH to 3.0 with 5 M HCl. A solution of pepsin in SGF (150 mg of pepsin in 10 mL SGF,60 000 U/mL) was prepared, and 667 μL of this solution was added to each sample. The volume was adjusted to 20 mL with ultrapure water. The samples were maintained under simulated gastric conditions for 2 h (37° C and 85 rpm in a rocker shaker). To initiate the intestinal phase, the samples were cooled on ice and mixed with 8 mL of previously warmed simulated intestinal fluid (SIF) electrolyte stock solution (pH 7.0), 3 mL of bile solution (10 mM), and incubated for 30 min at 37° C in a rocker shaker mixer (85 rpm). Then, 40 μL of 0.3 M CaCl_2_ (H_2_O)_2_ and 5 mL of trypsin (100 U/mL) were added to the mixture. The final volume was settled to 40 mL with water. The pH was adjusted to 7.0 with 5 M NaOH, and the samples were maintained under simulated small intestinal conditions for 2 h in similar conditions as in the previous phases. After this period, the digesta were cooled on ice and the samples were transferred to centrifuge tubes and centrifuged at 4° C for 45 min at 15157 g to separate the mixed micelles. The aqueous fraction containing the mixed micelles was collected and filtered through a 0.20 μm nylon filter. Then, 10 mL of micellar fraction in triplicate from each digestion was frozen at −20°C for subsequent pigment extraction for not more than three days.

### Chlorophyll Extraction

The raw materials, vegetable puree (2.3 g) and virgin olive oil (10 g), were extracted following a portioning phase system between DMF and n-hexane as described by Mínguez-Mosquera & Garrido-Fernández.^33^ Next, chlorophylls were transferred from DMF to diethyl ether and later evaporated up to total dryness. Fruit juice (100 mL) was extracted with 10 mL of acetone and 100 mL of diethyl ether. The mixture was homogenized and transferred to a funnel with 400 mL of NaCl. After stirring and phase separation, the upper diethyl ether phase was evaporated up to total dryness. The dry residue was dissolved in acetone, 25 mL for the olive oil, 500 μL for the puree, and 1 mL for the juice, and analyzed by HPLC. The corresponding frozen mixed micelles were thawed, and the chlorophyll pigments were extracted by liquid extraction (LE method)^15^ with the sequential addition of 10 mL of acetone, 10 mL of diethyl ether, and 10 mL of NaCl 10% (w/v). The samples were next vortexed for 1 min and centrifuged at 2151*g* for 5 min. The diethyl ether layer was collected and transferred to a clean tube. The extraction with diethyl ether was repeated a total of three times (until no more color was extracted), and the combined diethyl ether fractions were dried under a stream of nitrogen and redissolved in 0.5 mL of acetone. The samples were directly analyzed by HPLC.

### Influence of Variables during the Chlorophyll *In Vitro* Bioaccessibility Assay

#### Influence of the Chlorophyll Extraction Method

The filtered micellar fractions obtained from each standard digestion were split into three 10 mL aliquots. One micellar fraction was extracted following the LE method described before. The other two micellar fractions were lyophilized (Telstar LyoQuest-55) until complete dryness. One sample was extracted following the freeze-dried acetone (FDA) protocol previously described:^4^ the lyophilized micellar fractions were moistened with 200 μL of distilled water and stirred for 5 min. An additional 200 μL of DMF was added to the mixture and stirred again for 5 min. Then, 1.6 mL of acetone was added and mixed in an ultrasonic bath (10 min, 720 W). After filtration, the solvent layer (nylon, 0.22 μm) was directly analyzed by HPLC. The second lyophilized sample was extracted using a freeze-dried ether (FDE) method that started moistening the dry residue with 400 μL of distilled water and stirring the mixture for 5 min. Additional 400 μL of DMF was added to the mixture and stirred again for 5 min. Then, 3.2 mL of acetone was added, and the mixture was mixed in an ultrasonic bath (10 min, 720 W). Then, 1.2 mL of DMF was added again, and the mixture was reimmersed in an ultrasonic bath (10 min, 720 W). Next, 2 mL of diethyl ether and 4 mL of 10% NaCl (w/v) were subsequently added, stirred in a vortex, and then mixed in an ultrasonic bath for an additional 10 min. Finally, the mixture was centrifuged (2151*g* for 5 min at 4 °C) and the diethyl ether phase was evaporated under a stream of nitrogen until complete dryness. The residue was dissolved in 200 μL of acetone, filtered (0.20 μm), and directly analyzed by HPLC.

#### Influence of the Stirring Method

Three different mixing mechanisms were tested, a rocker shaker (as the standard condition), a vertical (Ver) shaker (Multirotator PTR-35, Grant bio), both at 85 rpm, and a vortex shaker (Multi Reax, Heidolph) at 1000 rpm. Each food was assayed three times following the standard conditions. Each time, one of the three different mixing mechanisms was utilized through the assay.

#### Influence of the Filtering Step

Each food was digested following the standardized protocol, in triplicate and at the end of the intestinal phase, 40 mL of each digesta were divided into two 20 mL of samples to separate the mixed micelles. After centrifugation, one sample (15 mL) was filtered (F) as explained before and the other 15 mL were directly frozen, without filtering (NF).

#### Influence of the Ultracentrifugation or Centrifugation

After the standard *in vitro* digestion process of each food in triplicate, the digesta were split into two samples of 6 mL (the volume was limited by the ultracentrifuge tubes). One of the digesta was centrifuged as explained before, and the other digesta were ultracentrifuged at 50 000*g* for 90 min (Optima MAX Ultracentrifuge, Beckman Coulter, rotor MLA-80). After centrifugation, the mixed micelles were filtered and frozen as described.

#### Influence of Gastric Lipase Utilization during the *In Vitro* Digestion

Each food (in triplicate) was assayed two times following the standard method described earlier. One of the experiments was run exactly the same as described before (without gastric lipase), but in the second experiment, the oral bolus was mixed with 0.48 mL of gastric lipase (60 U/mL) at the same time as pepsin addition during the gastric phase. The rest of the assays proceeded as the standard methodology.

### Chlorophyll Separation, Identification, and Quantification

Separation was carried out with an HPLC Hewlett-Packard HP 1100 by a reversed phase using a Mediterranea Sea18 column (200 mm × 4.6 mm, 3 μm particle size, Teknokroma, Barcelona, Spain) protected by the same material guard column (10 mm × 4.6 mm). The elution gradient was previously described^34^ with the mobile phases: (A) water/0.05 M ammonium acetate/methanol (1/1/8, v/v/v) and (B) methanol/acetone (1/1, v/v). The UV–vis spectra were recorded from 350 to 800 nm, although a sequential detection was performed at 410, 430, 450, and 666 nm. Data were collected and processed with an LC HP ChemStation (Rev.A.05.04). The identification of the chlorophyll compounds was made based on co-chromatography with authentic samples (commercial standards and laboratory-produced chlorophylls but previously identified by MS/MS) and from their spectral characteristics.^31,32^ Quantification of chlorophylls was performed with the corresponding calibration curves obtained by least-squares linear regression analysis over a concentration range, according to the quantities present in the analyzed samples (*R*^2^ > 0.999).

### Statistical Analysis

All of the experiments were carried out in triplicate, and data were expressed as means ± standard deviation (SD). The data were analyzed for differences among means using a one-way analysis of variance (ANOVA). Tukey′s multiple-range test was used as a post hoc comparison of statistical significance (*p* < 0.05). The statistical studies were carried out with OriginPro 2020b software.

## Results and Discussion

### Chlorophyll Profile in Raw Material and after *In Vitro* Digestion

**Figure 1:**
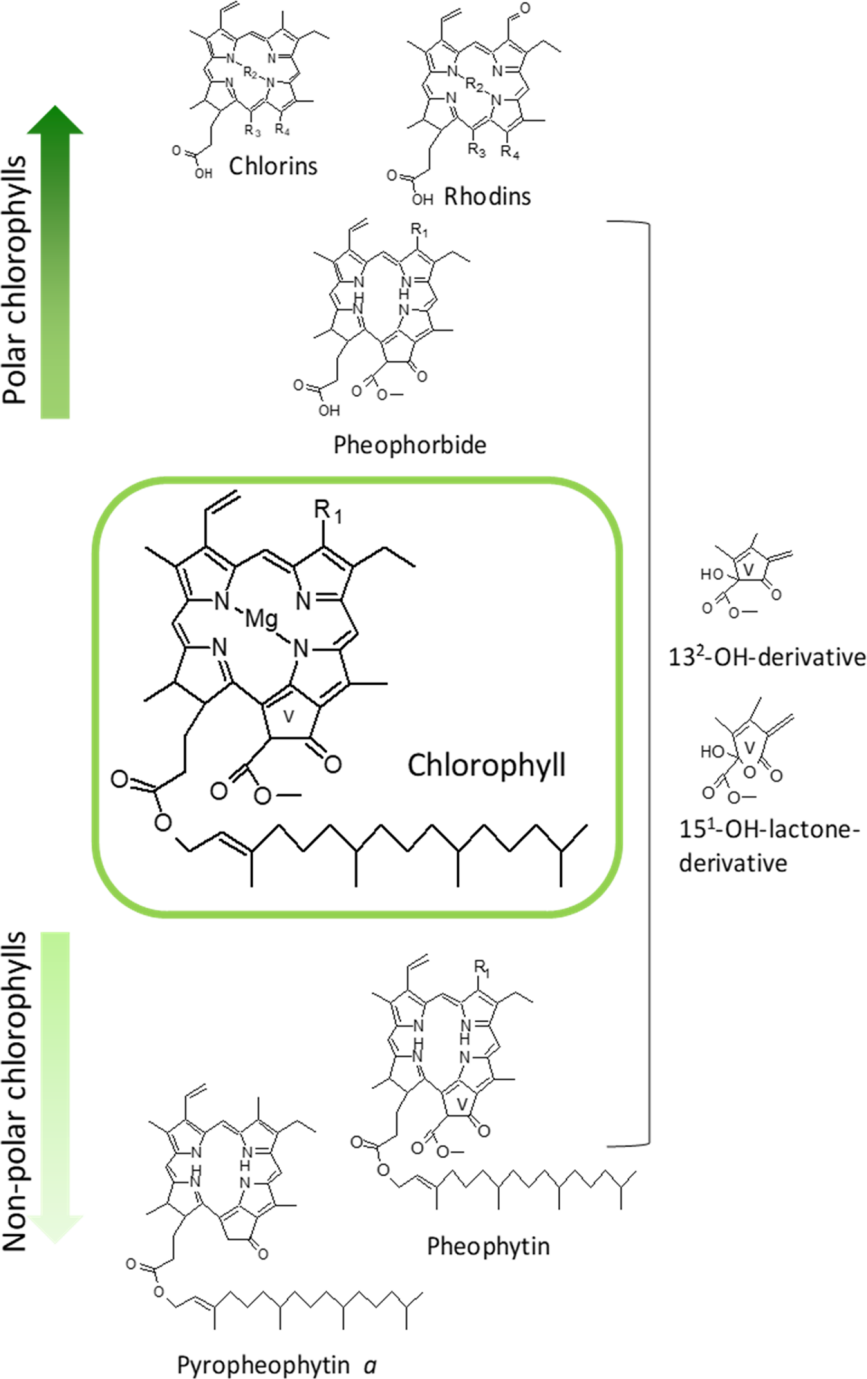
Chlorophyll structures present in the mixed micelles formed during the *in vitro* digestion of a vegetal puree, fruit juice, and virgin olive oil.

Food structure determines the amount and dynamics of nutrient uptake,^35^ as it influences key mechanisms, such as food comminution, food mixing, gastric kinetics, *etc*.^36^ There is increasing evidence that the food matrix plays an important role in the digestion and bioaccessibility of certain phytochemicals, such as polyphenols, anthocyanins, and carotenoids.^11,37,38^ Moreover, it has been shown that three of the main components that may interfere with phytochemicals digestion are water, fiber, and fat.^38,39^ For that reason, three food matrices with different structural characteristics: liquid (fruit juice), high fiber (vegetable puree), and high fat content (virgin olive oil) were selected. This strategy seeks to analyze the influence of the food matrix on the adaptation of the protocol. In addition, the food selection was made looking for foods with a chlorophyll composition as different as possible to study the impact of the digestion variables analyzed on structurally different chlorophylls. Pheophytins, pheophorbides, pyropheophytins, 13^2^-hydroxy-chlorophylls, and 15^1^-hydroxy-lactone-chlorophyll derivatives (Figure 1) are chlorophylls formed during the natural ripening/senescence of fruit and vegetables and also during the processing or storing of foods. But chlorins (*a* series) and rhodins (*b* series) with a characteristic opened isocyclic ring are usually present in foods due to the addition of the natural authorized food colorants E140 and E141.^40^ Therefore, all of the chlorophyll compounds analyzed in the present research are daily present in our diet. For example, the fruit juice (Table 1) showed a chlorophyll profile mainly formed by pheophorbides and pheophytins at a similar level, and also a significant presence of chlorins and rhodins (Figure 1). On the contrary, in the vegetable puree, more than 80% of the chlorophylls were pheophytins, mainly pyropheophytins, which are formed by the loss of the carboxymethoxy group at C13^2^ (Figure 1)^4^ and entail the most nonpolar chlorophyll derivatives. This higher quantity of pyro compounds is due to the sterilization process of the puree since heat treatment has shown to enhance the formation of these derivatives.^41^ Virgin olive oil exhibited a profile dominated also by pheophytins but with 25% of intact chlorophylls.^42^

**Table 1:** Chlorophyll Composition (Percentage) in Vegetable Puree, Fruit Juice, and Virgin Olive Oil (Raw Material) and the Mixed Micelles Obtained after the *In Vitro* Digestion of the Respective Foods with the Optimized Conditions

	fruit juice		vegetable puree		olive oil	
	raw material	micellar fraction	raw material	micellar fraction	raw material	micellar fraction
chlorins + rhodins	24.32	13.73	1.66	1.60	0.00	0.00
pheophorbides	32.71	18.02	17.06	26.27	0.01	0.00
chlorophylls	0.00	0.00	0.00	0.00	25.22	1.94
pheophytins	42.97	68.25	81.28	72.14	74.76	98.05
13^2^-OH-chls^1^	3.37	18.35	6.41	5.71	4.29	8.01
15^1^-OH-lactone-chls^2^	1.20	8.65	0.00	3.83	1.45	5.57
pyropheophytins	3.20	16.66	67.66	59.50	2.57	13.90

1 13^2^-OH-chls: stands for 13^2^-OH-pheophorbides, 13^2^-OH-chlorophylls, and 13^2^-OH-pheophytins.

2 15^1^-OH-lactone-chls stands for 15^1^-OH-lactone-pheophorbides and 15^1^-OH-lactone-pheophytins.

Table 1 shows the chlorophyll composition of the micelles formed after the *in vitro* digestion of the foods with the optimized conditions. As expected, during the chlorophyll digestion and micellarization, the main reaction was due to the acidic conditions of the gastric digestion phase.^14^ For example, in olive oil, the simulated digestion caused almost the complete conversion of chlorophylls into their Mg-free derivatives, pheophytins, as in all of the food matrices tested up to now.^1^ Another significant event was the formation of 15^1^-hydroxy-lactone and 13^2^-hydroxy derivatives formed due to oxidizing ambient attained during the *in vitro* digestion process that favors isocyclic ring oxidation and previously observed in other food matrices.^4,18^ The main derivative found after *in vitro* digestion in all cases was pheophytin, followed by pheophorbides, which is consistent with the previous results.^4,15^ Particularly interesting was the behavior of pyropheophytins, which showed an interesting high bioaccessibility degree, probably due to their lipophilic character, which could facilitate the incorporation into the corresponding micelles.

### Influence of the Extraction Method on Chlorophyll Bioaccessibility

One of the main drawbacks when working with chlorophylls is to guarantee the total extraction from the tissue without any artificial modification. Two main issues should be taken into account, the broad polarity of chlorophyll compounds and the characteristics of the matrix we are working with. In this line, organic solvent extraction has been the classical method for pigment extraction for years.^19^ Two different approaches have been developed to extract chlorophylls from micellar fractions. The first one is a direct solvent extraction method from fresh micellar fractions^14−17,43−46^ with the difficulty of the great amount of water accumulated in the sample at the end of the chlorophyll extraction. To solve this problem, a second approach is to introduce a freeze-drying step before the solvent extraction.^4,18,19^ To identify the best methodology, three different extracting protocols have been compared after the digestion of the different food matrices. A liquid-extraction (LE) method is an immediate solvent extraction^15^ previously used with vegetable matrices and a freeze-drying acetone protocol (FDA) is a solvent extraction from lyophilized samples applied to seaweeds.^4^ As the last method only uses acetone, we have modified the FDA method, introducing more range of polarity in the solvents used, trying to improve the extraction capacity (freeze-drying ether (FDE) method).

In liquid samples (Table 2), based on the total amount of chlorophylls, LE extraction was significantly better than freeze-dried (FD) methodologies (*p* < 0.05), which showed statistically similar levels between them. Even more, only LE extraction was able to extract polar chlorophylls (pheophorbides) and FDA could only recover pheophytin *a*. In this sense, FDE extraction improved FDA extraction as chlorins, pyropheophytins, and oxidized pheophytins were only extracted with FDE methodology. In terms of chlorophyll profile, FDE was relatively similar to LE, but the total amount recovered was much lower. A similar trend, although much more smooth, could be observed when fiber-rich foods were assayed (Table 2). LE methodology extracted significantly higher amounts (*p* < 0.05) of chlorophylls from vegetable puree′s micellar fraction than the FDA method. Interestingly, starting from high-fiber foods, LE and FDA were equivalents in polarity extraction capacity, as the chlorophyll profile in micellar fractions was similar with both methodologies. On the contrary, FDE exhibited a lower extraction capacity with polar chlorophyll compounds and higher with nonpolar chlorophylls (such as pheophytins and pyropheophytins). However, when fat-rich foods were digested (Table 2), the three assayed methods were equivalents, as all extracted the same amounts of chlorophylls from micellar fractions (*p* < 0.05) and were equally effective to recover polar and nonpolar chlorophylls.

**Table 2:** Chlorophyll Composition (Percentage) and Total Chlorophylls (mg/kg) in the Mixed Micelles Obtained after the *In Vitro* Digestion of Vegetable Puree, Fruit Juice, and Virgin Olive Oil Extracted with Different Protocols

	fruit juice			vegetable puree			virgin olive oil		
	LE^1^	FDA^2^	FDE^3^	LE	FDA	FDE	LE	FDA	FDE
chlorin + rhodin	14.21^a^	0.00^b^	6.75^a^	0.74^a^	1.35^a^	1.64^a^	0.00^a^	0.00^a^	0.00^a^
pheophorbide *b*	5.77^a^	0.00^b^	0.00^b^	0.00^a^	0.00^a^	0.00^a^	0.00^a^	0.00^a^	0.00^a^
pheophorbide *a*	9.42^a^	0.00^b^	0.00^b^	24.94^a^	22.33^b^	1.74^c^	0.00^a^	0.00^a^	0.00^a^
chlorophyll *b*	0.00^a^	0.00^a^	0.00^a^	0.00^a^	0.00^a^	0.00^a^	1.03^a^	4.27^b^	1.39^a^
chlorophyll *a*	0.00^a^	0.00^a^	0.00^a^	0.00^a^	0.00^a^	0.00^a^	0.00^a^	0.00^a^	0.92^b^
pheophytin *b*	9.98^a^	0.00^b^	20.83^a^	20.15^a^	20.30^b^	25.96^a^	0.00^a^	0.00^a^	0.00^a^
pheophytin *a*	60.61^a^	100.00^b^	72.42^c^	53.89^a^	55.71^a,b^	70.41^b^	98.97^a^	95.73^a^	97.69^a^
13^2^-OH-chls^4^	18.67^a^	0.00^b^	24.18^c^	6.55^a^	8.34^a^	0.56^b^	7.24^a^	7.33^a^	5.93^a^
15^1^-OH-lactone-chls^4^	6.45^a^	0.00^b^	3.95^c^	0.00^a^	0.00^a^	0.00^a^	3.22^a^	2.85^a^	1.82^a^
pyropheophytin *a*	15.80^a^	0.00^b^	35.53^a^	63.77^a^	64.41^b^	81.46^a^	14.63^a^	8.01^a^	13.52^a^
total chlorophylls^5^	0.17^a^	0.07^b^	0.07^b^	29.88^a^	19.22^b^	21.00^a,b^	10.36^a^	9.99^a^	9.44^a^
SD	0.01	0.01	0.01	2.25	1.83	1.60	1.05	1.08	1.11

1 LE: liquid extraction.

2 FDA: freeze-drying acetone.

3 FDE: freeze-drying ether.

4 As in Table 1.

5 Total chlorophylls stand as the sum of chlorin, rhodin, pheophorbides, chlorophylls, and pheophytins. Superscripts describe the significance (on absolute amount) of the difference between the extraction methods within each chlorophyll compound for each food.

Altogether, our data demonstrate that the food matrix determines the development of the chlorophyll extraction method from mixed micelles. For example, FD methodology was the best option when analyzing the digestion of seaweeds, as the cell wall and extracellular material of macroalgae made the solvent extraction from fresh micellar fractions difficult.^4^ This protocol has been successfully applied for microalgae^18^ and orange peels.^19^ Taking into account the data in Table 2 and to select the same extraction method for all of the samples, LE should be the method of election for extraction standardization of micelles from juice, puree, and olive oil. However, research on chlorophyll bioaccessibility from a new food matrix will require further testing to elucidate which extraction method is the best option. In this sense, more sustainable methods should also be included as the application of eutectic and ionic liquids.^19^

### Influence of the Type of Mixing on Chlorophyll Bioaccessibility

As it has been recently stated^21^ in the INFOGEST 2.0 protocol, standardization of the shaking method is vital and should be carefully considered. However, independent of the *in vitro* digestion protocol assayed, a large variety of mixing methods have been used for chlorophyll *in vitro* digestion in the literature. Initially, magnetic stirring in a water bath was the preferred option at different speeds: 95,^17,43^ 150 rpm,^46^ and even 500 rpm.^15,16^ More recently, different types of shakers have been employed,^18,44^ including the horizontal shaker,^4^ rocker shaker,^19^ or reciprocal shaker,^17^ originating a diversity of results. This makes it necessary to perform a comparative study to clarify the most suitable conditions for chlorophyll *in vitro* digestion and, therefore, three different types of mixing currently used with other phytochemicals have been tested here: vertical, rocker, and vortex shaker.

For juice and puree matrices (Table 3), the agitation with vortex allowed a statistically significant (*p* < 0.05) higher chlorophyll micellarization than with the vertical shaker, but similar to the rocker shaker (*p* < 0.05). When assaying juicy matrices, the chlorophyll profile with the three homogenization methods was almost the same, which implies that the type of agitation does not introduce any significant artificial modification in the chlorophyll profile, except for the high amount of 13^2^-hydroxy-chlorophylls micellarizated with the vortex shaker. On the contrary, when working with high-fiber foods, besides a statistically similar chlorophyll amount as when the vortex is used, the rocker shaker showed an improved capacity for pheophorbide micellarization compared to the other two mixing methods. Finally, for the oily matrix, the vortex shaker also seemed to favor chlorophyll micellarization (statistically significant compared to the rocker shaker *p* < 0.05), while the other two agitation methods were similar. Anyhow, the chlorophyll profile in the micelles was exactly the same.

**Table 3:** Chlorophyll Composition (Percentage) and Total Chlorophylls (mg/kg) in the Mixed Micelles Obtained after the *In Vitro* Digestion of Vegetable Puree, Fruit Juice, and Virgin Olive Oil Homogenized with Different Mixing Models

	fruit juice			vegetable puree			virgin olive oil		
	vertical	rocker	vortex	vertical	rocker	vortex	vertical	rocker	vortex
chlorin + rhodin	14.33^a^	15.47^a^	14.21^a^	1.26^a^	1.03^a^	1.02^a^	0.00^a^	0.00^a^	0.00^a^
pheophorbide *b*	7.87^a^	8.49^a^	5.77^a^	0.00^a^	0.00^a^	0.00^a^	0.00^a^	0.00^a^	0.00^a^
pheophorbide *a*	9.50^a^	10.06^a^	9.42^a^	23.11^a^	31.23^b^	24.94^a,b^	0.00^a^	0.00^a^	0.00^a^
chlorophyll *b*	0.00^a^	0.00^a^	0.00^a^	0.00^a^	0.00^a^	0.00^a^	1.33^a^	1.86^a^	1.03^a^
chlorophyll *a*	0.00^a^	0.00^a^	0.00^a^	0.00^a^	0.00^a^	0.00^a^	0.00^a^	0.00^a^	0.00^a^
pheophytin *b*	13.76^a^	8.23^a^	9.98^a^	20.41^a^	16.21^b^	20.15^a^	0.00^a^	0.00^a^	0.00^a^
pheophytin *a*	54.54^a^	57.75^a^	60.61^a^	55.13^a^	51.60^a^	53.89^a^	98.67^a^	98.14^a^	98.97^a^
13^2^-OH-chls^1^	11.87^a^	15.34^a^	18.67^b^	5.99^a^	8.84^b^	6.55^a^	4.37^a^	3.37^a^	3.24^a^
15^1^-OH-lactone-chls^1^	3.80^a^	7.55^a^	6.45^a^	0.00^a^	0.00^a^	0.00^a^	3.18^a^	5.15^a^	3.22^a^
pyropheophytin *a*	24.63^a^	18.54^a^	15.80^a^	64.20^a^	58.87^a^	63.77^a^	9.57^a^	14.80^a^	17.13^b^
total chlorophylls^2^	0.12^a^	0.16^b^	0.19^b^	23.56^a^	28.62^b^	29.88^b^	14.67^a^	11.19^a^	18.54^b^
SD	0.01	0.01	0.02	2.09	2.30	2.25	1.20	1.10	1.51

1 As in Table 1.

2 As in Table 2. Superscripts describe the significance (on absolute amount) of the difference among mixing methods within each chlorophyll compound for each food.

The objective of the *in vitro* digestion protocols is to mimic the physiological conditions as much as possible. At present, a growing trend is to reduce the mixing speed.^47^ Although in our comparative study, the vortex shaker was able to micellarizate higher amounts of chlorophylls in all of the food matrices, we did not consider it as the best option in any case. To be able to reach a proper mixing, the vortex shaker had to be set up at 1000 rpm, while the other two shakers were set up at 85 rpm. In addition, vortex mixers create vortex and circulation loops when shaking,^48^ complicating the mixing among the digestion components, besides the excessive aeration that takes this shaking method far away from physiological conditions. Although it is complicated to reflect the exact mixing of gastric and intestinal content *in vivo*,^36^ probably the rocker shaker is the method that better mimics physiological conditions, as the movement allows the best exposition of all substrates and enzymes gently. Therefore, we consider that this could be the best option for chlorophyll digestion.

### Influence of Centrifugation in the Composition of Chlorophyll-Rich Mixed Micelles

Lipophilic metabolites must be included in mixed micelles stabilized by bile salts prior to their intestinal absorption, in which a centrifugation step is necessary to separate the aqueous phase from the pellet and, if existing, oily supernatant. First assays considered the ultracentrifugation as the best option to separate the micellar fraction, ranging from 50 000 to 167 000 g.^4,14−16^ But the most recent research studies have considerably reduced the centrifugation speed (between 4255 and 15 000 g) and time (60–20 min).^17,19,44^ To the best of our knowledge, no research has been made to understand the significance of the centrifugation speed in the micellarization of chlorophylls. There is only a pilot study^49^ where ultra and centrifugation are compared regarding the incorporation of carotenoids into micelles with no significant differences. Therefore, we have analyzed the influence of this variable in the incorporation of chlorophylls from the three mentioned food matrices into micelles (Table 4). In juicy and oily matrices, the total amount of chlorophylls incorporated into the mixed micelles ultracentrifuged is almost half of the chlorophylls incorporated into the micelles when centrifuged at a lower speed (*p* < 0.05). Interestingly, ultracentrifugation could have favored the sedimentation of higher, thicker micelles rich in chlorophylls in juicy and oily matrices and, therefore, it should be considered as a critical factor when determining chlorophyll bioaccessibility. However, in fiber-rich food, the speed of centrifugation did not interfere with the inclusion of chlorophylls in the mixed micelles (*p* < 0.05). The interaction of fiber with other meal components during digestion could affect the rheological and colloidal state of digesta.^50^ For example, fiber could increase digesta viscosity, which impairs the efficiency of the emulsification, producing smaller lipid droplets^51^ that will not precipitate, even at high speed as during ultracentrifugation. In addition, it has been shown that fiber also stabilizes the lipid emulsions through a complex interplay, not fully understood at present,^50^ which could hamper their sedimentation.

**Table 4:** Chlorophyll Composition (Percentage) and Total Chlorophylls (mg/kg) in the Mixed Micelles Obtained after the *In Vitro* Digestion of Vegetable Puree, Fruit Juice, and Virgin Olive Oil Isolated by Ultracentrifugation (50 000*g*) and Centrifugation (15 000*g*)

	fruit juice		vegetable puree		virgin olive oil	
	centrifugation	ultracent.^1^	centrifugation	ultracent.	centrifugation	ultracent.
chlorin + rhodin	15.00^a^	15.00^a^	2.82^a^	2.87^a^	0.00^a^	0.00^a^
pheophorbide *b*	6.84^a^	7.98^a^	0.00^a^	0.00^a^	0.00^a^	0.00^a^
pheophorbide *a*	9.92^a^	15.58^a^	25.89^a^	25.19^a^	0.00^a^	0.00^a^
chlorophyll *b*	0.00^a^	0.00^a^	0.00^a^	0.00^a^	2.19^a^	3.26^a^
chlorophyll *a*	0.00^a^	0.00^a^	0.00^a^	0.00^a^	0.00^a^	0.00^a^
pheophytin *b*	4.29^a^	7.09^a^	16.00^a^	15.14^a^	0.00^a^	0.00^a^
pheophytin *a*	63.96^a^	54.35^b^	55.30^a^	56.79^a^	97.8^a^	96.7^a^
13^2^-OH-chls^2^	18.38^a^	24.32^a^	4.04^a^	4.43^a^	11.57^a^	10.56^a^
15^1^-OH-lactone-chls^2^	10.25^a^	13.64^a^	8.22^a^	9.59^a^	6.01^a^	7.13^a^
pyropheophytin *a*	17.02^a^	11.94^b^	56.21^a^	53.02^a^	10.54^a^	9.81^a^
total chlorophylls^3^	0.17^a^	0.11^b^	23.80^a^	22.30^a^	12.66^a^	7.74^b^
SD	0.02	0.01	2.22	0.69	1.50	0.64

1 Ultracentrifugation.

2 As in Table 1.

3 As in Table 2. Superscripts describe the significance (on absolute amount) of the difference between centrifugation speeds within each chlorophyll compound for each food.

However, it is important to analyze not only the total amount of chlorophylls in mixed micelles but also the chlorophyll profile to detect if the centrifugation speed influences the inclusion of chlorophylls with different polarities. Indeed, ultracentrifugation made the inclusion of nonpolar chlorophylls in a liquid matrix difficult, where the lower amounts of pheophytin and pyropheophytin in mixed micelles isolated through ultracentrifugation compared to centrifugation are statistically significant (*p* < 0.05). In a highly polar ambient, as an aqueous juice matrix, the favoring inclusion of very nonpolar chlorophylls (pheophytins and pyropheophytins) in the mixed micelles is understandable, and the higher centrifugation times tend to balance polarity through the sedimentation of nonpolar micelles in a highly polar medium. Anyhow, when working with very polar matrices, the centrifugation speed is a determinant factor to take into account to avoid the affected results. The morphology and structure of mixed micelles have been recently analyzed by scattering techniques and atomistic simulations.^52^ Larger micelles are formed with long fatty acids than with short fatty acids. It is then possible that larger micelles could be formed with nonpolar chlorophylls that carry a phytol chain (C_20_H_20_), which is not present in polar chlorophylls (pheophorbides).

### Influence of Filtering on Chlorophyll Bioaccessibility

It is assumed that after the centrifugation phase, a filtration step with a 0.20 μm filter pore for hydrophilic solutions (cellulose acetate or nylon) is mandatory.^4,14,17,19,44^ Such a protocol is not only imposed when working with chlorophylls but also with carotenoids.^23^ The objective of such a procedure is to remove other interfering aggregates. As stated,^23^ filtration should be tested to discern the implications of such protocol, but at present, it has not been performed for chlorophylls (Table 5).

**Table 5:** Chlorophyll Composition (Percentage) and Total Chlorophylls (mg/kg) in the Mixed Micelles Obtained after the *In Vitro* Digestion of Vegetable Puree, Fruit Juice, and Virgin Olive Oil, Filtered (F), and Nonfiltered (NF)

	fruit juice		vegetable puree		virgin olive oil	
	F^1^	NF^2^	F	NF	F	NF
chlorin + rhodin	10.25^a^	9.07^a^	1.55^a^	0.80^a^	0.00^a^	0.00^a^
pheophorbide *b*	7.10^a^	7.04^a^	0.00^a^	0.00^a^	0.00^a^	0.00^a^
pheophorbide *a*	14.46^a^	15.30^a^	19.02^a^	12.53^a^	0.00^a^	0.00^a^
chlorophyll *b*	0.00^a^	0.00^a^	0.00^a^	0.00^a^	2.69^a^	2.67^a^
chlorophyll *a*	0.00^a^	0.00^a^	0.00^a^	0.00^a^	0.00^a^	0.00^a^
pheophytin *b*	0.00^a^	0.00^a^	20.38^a^	22.15^a^	0.00^a^	0.00^a^
pheophytin *a*	62.65^a^	60.44^a^	55.04^a^	64.53^b^	97.30^a^	97.32^a^
13^2^-OH-chls^3^	20.99^a^	25.92^a^	0.80^a^	1.13^a^	13.86^a^	11.50^a^
15^1^-OH-lactone-chls^3^	10.37^a^	15.83^a^	4.30^a^	4.02^a^	7.92^a^	8.26^a^
pyropheophytin *a*	15.22^a^	16.68^a^	59.13^a^	71.04^b^	15.33^a^	13.61^a^
total chlorophylls	0.16^a^	0.14^a^	27.51^a^	25.03^a^	10.50^a^	11.35^a^
SD	0.02	0.02	1.63	1.70	0.60	1.06

1 F: filter.

2 NF: no filter.

3 As in Table 1.

4 As in Table 2. Superscripts describe the significance (on absolute amount) of the difference between filtering/nonfiltering within each chlorophyll compound for each food.

In terms of the total amount of micellarized chlorophylls, the data confirmed that filtration does not introduce any modification in the selection of the micelle size, as the total amounts of chlorophylls incorporated into the mixed micelles were the same (*p* < 0.05) independent of the food matrix. Also, the analyzed micelles, filtrated or not, contained a similar chlorophyll profile, which means that the filtration did not retain any micelle by polarity with a clear exception. When chlorophyll-rich mixed micelles originated during the digestion of fiber-rich (puree) food are filtered, a reduction on nonpolar chlorophyll-rich micelles (pheophytins and pyropheophytins) was produced. The filter used for micelle filtration was a nylon filter, as a polar solution was being filtered. However, as stated before, one of the main drawbacks when working with chlorophylls is the broad spectrum of polarity that chlorophylls exhibit. Mixed micelles are formed with highly polar chlorophylls (as pheophorbides) and very nonpolar chlorophylls (as pyropheophytins). Commonly, the presence of pyropheophytins in the raw material to be digested is scarce; however, to check all of the possible interferences during the *in vitro* digestion of chlorophylls and to force the methodology, a food where the presence of pyropheophytins was more than 65% of the total chlorophyll profile (Table 1) was selected. Therefore, when micelles contained a normal amount of pyropheophytins (around 15%), filtration did not affect the chlorophyll profile. On the contrary, when pyropheophytins domain the chlorophyll profile of the mixed micelles, the filter is going to retain the most nonpolar micelles (with abundant pyropheophytins). In conclusion, although filtration is recommended after digestion and before entering the HPLC equipment, it should be avoided when analyzing foods rich in pyropheophytins.

### Influence of Gastric Lipase

In routinely *in vitro* assays, the inclusion of an additional gastric lipase besides the pancreatic one is avoided, mainly for economic reasons. However, the lipid digestion starts in the stomach with a human gastric lipase (HGL), which is responsible for up to 25% of the triacylglycerides present in an emulsified meal.^21^ At present, different commercial enzymes are available: recombinant HGL, dog and rabbit gastric lipase, and microbial lipases. Different comparative studies have been developed,^53^ depending on the origin, and the enzymes showed different stereospecificity in the triacylglycerol (TAG) hydrolysis, pH stability, *etc*. Following the INFOGEST 2.0 protocol, we have tested the rabbit GL during the simulated *in vitro* digestion of the three foods to analyze the influence on chlorophyll bioavailability.

As can be seen from the results obtained (Table 6), the addition of gastric lipase did not modify the chlorophyll composition of mixed micelles formed during the digestion of fruit juice or vegetable puree. In the end, the proportion of fats in both foods was less than 0.5% as described in Materials and Methods, and the fixed amount of pancreatic lipase added at the simulated digestion, following the INGOGEST 2.0 protocol, seemed to be more than enough to complete the lipolysis in both samples. Nonetheless, the results were completely different when the olive oil was digested with a gastric lipase, obtaining approximately half of the total chlorophylls incorporated into micelles. As it could be expected, when incorporating a gastric lipase to the digestion of an oily food (with almost 100% of TAGs), the reaction of lipolysis increased during the digestion, where a less amount of TAGs for the micelle formation is available. Consequently, the fewer the amount of micelles could be formed, the fewer the chlorophyll compounds could be incorporated into them. In conclusion, while for nonfatty foods, the presence of a gastric lipase is not necessary, when assaying fat-rich food matrices, the addition of a gastric lipase is essential to reproduce the physiological digestion and micellarization of chlorophylls as much as possible.

**Table 6:** Chlorophyll Composition (Percentage) and Total Chlorophylls (mg/kg) in the Mixed Micelles Obtained after the *In Vitro* Digestion of Vegetable Puree, Fruit Juice, and Virgin Olive Oil, Including Rabbit Gastric Lipase (+ RGL) or not (− RGL)

	fruit juice		vegetable puree		virgin olive oil	
	+RGL	–RGL^1^	+RGL	–RGL	+RGL	–RGL
chlorin + rhodin	10.50^a^	13.73^a^	1.87^a^	1.60^a^	0.00^a^	0.00^a^
pheophorbide *b*	9.31^a^	7.05^a^	0.00^a^	0.00^a^	0.00^a^	0.00^a^
pheophorbide *a*	12.86^a^	10.97^a^	25.03^a^	26.27^a^	0.00^a^	0.00^a^
chlorophyll *b*	0.00^a^	0.00^a^	0.00^a^	0.00^a^	2.55^a^	1.94^a^
chlorophyll *a*	0.00^a^	0.00^a^	0.00^a^	0.00^a^	0.00^a^	0.00^a^
pheophytin *b*	5.82^a^	7.01^a^	20.22^a^	18.19^a^	0.00^a^	0.00^a^
pheophytin *a*	61.50^a^	61.24^a^	52.88^a^	53.96^a^	97.45^a^	98.05^a^
13^2^-OH-chls^2^	15.35^a^	18.35^a^	3.58^a^	5.71^a^	7.46^a^	8.01^a^
15^1^-OH-lactone-chls^2^	9.91^a^	8.65^a^	4.61^a^	3.83^a^	6.37^a^	5.57^a^
pyropheophytin *a*	13.77^a^	16.66^a^	60.57^a^	59.50^a^	13.32^a^	13.90^a^
total chlorophylls^3^	0.15^a^	0.17^a^	26.43^a^	28.23^a^	6.50^a^	10.88^b^
SD	0.01	0.01	0.04	1.87	0.21	0.73

1 the same experiment as in Table 1.

2 As in Table 1.

3 As in Table 2. Superscripts describe the significance (on absolute amount) of the difference between the *in vitro* digestion with and without rabbit gastric lipase within each chlorophyll compound for each food.

All in all, the present study analyzes the variables that can interfere during the determination of the *in vitro* bioaccessibility of chlorophylls and develop a protocol that takes into account the complexity and variability of different chlorophylls. Anyhow, as stated previously, although *in vitro* models cannot substitute *in vivo* assays, they should mimic physiological conditions as much as possible. Avoiding artificial alterations during the *in vitro* digestion protocol will bring both approaches closer, allowing *in vitro* models to reproduce the *in vivo* metabolism better.
